# Pegylated arginine deiminase synergistically increases the cytotoxicity of gemcitabine in human pancreatic cancer

**DOI:** 10.1186/s13046-014-0102-9

**Published:** 2014-12-12

**Authors:** Rouzbeh Daylami, Diego J Muilenburg, Subbulakshmi Virudachalam, Richard J Bold

**Affiliations:** Department of Surgery, University of California, Davis Medical Center, Sacramento, CA USA; Division of Surgical Oncology, Suite 3010, University of California, Davis Cancer Center, 4501 X Street, Sacramento, CA 95817 USA

**Keywords:** PEG-ADI, Pancreatic cancer, RRM2, Gemcitabine

## Abstract

**Background:**

Pancreatic ductal adenocarcinoma has proven to be one of the most chemo-resistant among all solid organ malignancies. Several mechanisms of resistance have been described, though few reports of strategies to overcome this chemo-resistance have been successful in restoring sensitivity to the primary chemotherapy (gemcitabine) and enter the clinical treatment arena.

**Methods:**

We examined the ability of cellular arginine depletion through treatment with PEG-ADI to alter in vitro and in vivo cytotoxicity of gemcitabine. The effect on levels of key regulators of gemcitabine efficacy (e.g. RRM2, hENT1, and dCK) were examined.

**Results:**

Combination of PEG-ADI and gemcitabine substantially increases growth arrest, leading to increased tumor response in vivo. PEG-ADI is a strong inhibitor of the gemcitabine-induced overexpression of ribonucleotide reductase subunit M2 (RRM2) levels both in vivo and in vitro, which is associated with gemcitabine resistance. This mechanism is through the abrogation of the gemcitabine-mediated inhibitory effect on E2F-1 function, a transcriptional repressor of RRM2.

**Conclusion:**

The ability to alter gemcitabine resistance in a targeted manner by inducing metabolic stress holds great promise in the treatment of advanced pancreatic cancer.

## Background

Traditional chemotherapy is cytotoxic to normal and malignant cells through the induction of apoptosis, however resistance to apoptotic cell death is a significant barrier to effective therapy in various cancers. Pancreatic ductal adenocarcinoma (PDAC) is the prototypical example of a tumor type resistant to the apoptotic effect of chemotherapy. Gemcitabine is the mainstay in the chemotherapeutic treatment of PDAC, though the clinical benefit is a prolongation of average survival by a minimal 6 weeks [1]. There has been a great deal of focus on the mechanisms that confer resistance to apoptosis in pancreatic cancer including aberrations in central mediators of apoptotic cell death. This has led to several investigations that have combined a variety of therapies directed at targets that may increase the sensitivity to gemcitabine [2–4]. In addition, there may be chemotherapy-specific mechanisms of resistance which can serve as targets for therapy to restore apoptotic cell death following chemotherapy exposure. Gemcitabine is administered as a pro-drug which must first be taken up by cells using the transport protein human equilibrative nucleoside transporter-1 (hENT1) [5]. It is then converted into an active metabolite by deoxycytidine kinase (dCK), allowing for incorporation into DNA as a nucleoside analog but structurally ending DNA synthesis. Ribonucleotide reductase subunit M2 (RRM2) converts the active metabolite of gemcitabine into an inactive form. Alterations of hENT-1, dCK and RRM2 have all been associated with resistance to gemcitabine-induced cell death in lung cancer as well as PDAC [6–9]. Therefore, therapies that specifically target the overexpression of any of these proteins altering gemcitabine metabolism may restore sensitivity to the apoptotic effect of gemcitabine, conferring a more significant clinical benefit to patients. Proof of this approach has been demonstrated by siRNA-mediated knockdown of RRM2 in colon and pancreatic cancer in vitro, which restored chemotherapeutic response to gemcitabine [10–12].

Although regulation of ribonucleotide reductase activity during cell cycle progression is well understood, the regulation of cellular level of the protein subunits is less well understood. While it has been frequently observed that RRM2 is upregulated following exposure to gemcitabine, the mechanism is unclear though various transcription factors including AP-1 and NF-κB have been implicated. RRM2 has been shown to be negatively regulated by the transcription factor E2F-1 [13,14]. Furthermore, the function of E2F-1 is regulated indirectly by arginine through the formation of a activated ternary complex of the arginine methyltransferase, PRMT2, the retinoblastoma (RB) protein and E2F-1[15]. Arginine also serves as a cellular store for nitric oxide, which further regulates the function of RB [16]. Therefore, through two discrete methods of regulation of RB function, arginine may potentially alter E2F-1 function and subsequent RRM2 levels. We have already demonstrated the majority of human PDAC tumors are deficient for argininosuccinate synthetase (ASS), the rate-limiting enzyme involved in arginine synthesis [17]. In this setting of ASS deficiency, PDAC are dependent on transmembrane amino acid transporters to maintain intracellular arginine levels. Pegylated arginine deiminase (PEG-ADI) has shown promise as a targeted therapeutic agent in the treatment of several types of cancer through its ability to decrease extracellular arginine, which limits the availability for cellular uptake [18–24].

Therefore, given the background potentially linking arginine metabolism to E2F-1 function, a known regulator of RRM2 expression, as well as the ability of PEG-ADI treatment to alter arginine metabolism, we sought to evaluate the effect of PEG-ADI on the biochemical and cellular response of PDAC to gemcitabine both in vitro and in vivo.

## Materials and methods

### Reagents and cell lines and culture

The pancreatic cancer cell lines MIA-PaCa2 and PANC-1 were purchased from American Type Culture Collection (Rockville, MD) while the L3.3 cell line was a generous gift from David McConkey (MDACC, Houston, TX); all cell lines were cultured in high glucose Dulbecco’s modified Eagle’s medium (GIBCO) supplemented with 10% fetal bovine serum, penicillin-streptomycin, sodium pyruvate, multivitamin and minimal non-essential amino acids (GIBCO). Pegylated arginine deiminase was generously provided by DesigneRx (Vallejo, CA) and gemcitabine was provided by our institutional pharmacy. All other chemicals were purchased from Sigma-Aldrich, Inc. unless otherwise stated. We have previously shown that the L3.3 cell line expresses ASS and that the MIA-PaCa-2 and PANC-1 cell lines are deficient in ASS [17].

### Determination of intracellular amino acid levels

In brief, following PEG-ADI therapy for 24 hours (1.0 μg/ml), cells are harvested, lysed and cytoplasmic content isolated following centrifugation (15,000 g × 15 min). Amino acid levels are quantitated in a 200 μl aliquot using the Beckman 6300 serum amino acid analyzer, which uses mass spectrometry to determine individual amino acid prevalence. Samples are spiked with AE-cysteine as an internal standard at a concentration of 3 nM to ensure quantitative accuracy. To determine the ability of cells to synthesize arginine, cells were washed and incubated in HBSS for 24 hours and then for an additional four hours in the absence or presence of citrulline and aspartate, the two substrates for ASS to synthesize arginine.

### MTT assay

96 well plates were seeded with 2.5 × 10^3^ cells per well and allowed to recover for 24 hours. Following treatment as described in results (eight wells per treatment group), MTT reagent (5 mg/mL) was added to each well and the plates incubated (37 C, 4% CO2) in the dark for 4 hours followed by incubation in 10% SDS overnight. Absorbance at 570 nM (reference filter 655 nM) was then measured using a microplate spectrophotometer (BioRad Laboratories). Each treatment was repeated three times. Statistical differences among treatment groups was determined by ANOVA with posthoc t-test.

### Western blot

Following treatment, cells were harvested with Trypsin 0.05% (GIBCO), washed with PBS and lysed with buffer (Cell Signaling Technology) containing 20 mM Tris (pH 7.5), 150 mM NaCl, 1 mM EDTA, 1 mM EGTA, 1% Triton X-100, 2.5 mM sodium pyrophosphate, 1 mM β-Glycerolphosphate, 1 mM Na_3_VO_4_, 1 μg/mL Leupeptin, and 1 mM PMSF. 75 to 100 μg of protein were loaded and electrophoretically separated by SDS gel then transferred to nitrocellulose by electrophoresis. The resulting blots were probed with primary antibodies and species specific secondary antibodies then developed by chemilumenscent technique. Antibodies used are as follows: RRM2, hENT-1, dCK and β-Actin (SantaCruz Biotechnology), Caspase3 (BD Pharmigen).

### Cell cycle analysis

6 well plates were seeded with 1×10^5^ cells and after a 24 hour recovery period were treated as described in results and then harvested with trypsin 0.05%. Cells were then washed with PBS and incubated in FacsMax (Genlantis Inc.) on ice followed by fixation in 70% ethanol. Prior to analysis, ethanol was removed and cells were resuspended in RNAse-A (200 μg/mL) at 37°C. After addition of an equal volume of propidium iodide (200 μg/mL) and incubation in dark, samples were assayed for PI content on FACScan flowcytometer (Beckton Dickinson) and analyzed by FlowJo software (Tree Star, Inc.). The sub-G1 population was defined as the percentage of cells with <2 N DNA content. All measurements were repeated in at least 3 independent experiments.

### Transfections and reporter assays

Transient transfections were carried out using the Lipofectin Reagent (Life Technologies, Inc., Gaithersburg, MD). Cells were cultured to 60% confluence in 6 × 35 mm well plates. They were serum starved for 24 hours. Cells were transfected with 2.5 mg of the 3× E2F-tk-luc plasmid (generous gift from Dr. Hong-Wu Chen, UC Davis) [25]. Transfections were carried out for 12 hours and then the medium was changed to serum containing medium. After an additional 12 hours, cells underwent treatment with either gemcitabine or PEG-ADI, or the combination. Cells were harvested within 60 hours of transfecton and lysed per the luciferase reporter protocol (Promega, Madison WI).Luciferase was assayed on a Monolight 2010 luminometer. Assays were normalized to μg of total cellular protein quantified using the BioRad reagent, and expressed as Relative Light Units (RLU) /μg protein. All experiments were performed in triplicate and data presented as mean +/- standard deviation.

### Annexin-V/PI flow cytometry

6 well plates were seeded with 1×10^5 cells and after a 24 hour recovery period were treated as described in results and then harvested with trypsin 0.05%. After washing and re-suspending cells in 200uL of PBS, 1 μL of AnnexinV-Cy5reagent (Biovision Inc.) and 1 μL of PI (1 mg/mL) were added and cells were incubated in dark for 15 minutes on ice then immediately analyzed on a Stratedigm S1400 flow cytometer (Stratedigm Inc.). Samples were analyzed by drawing quadrants around the healthy cell population in the negative control samples using FlowJo software. Apoptotic cells were taken to be the percentage of Annexin V positive cells. All measurements were repeated in at least 3 independent experiments.

### Mouse xenograft model

Six to eight-week old athymic mice were maintained in a dedicated Animal Care Facility according to institutional guidelines and fed an unrestricted mouse diet during the entirety of the experiment. Subcutaneous xenografts were seeded in both flanks of 4 mice per treatment group after suspending 1×10^6^ cells growth medium and diluting 1:1 (v/v) with Matrigel in a final volume of 200 μL. Tumors were allowed to reach a diameter of approximately 5 mm prior to initiating treatment. All drugs were diluted in PBS and injected intraperitoneally in a volume of 500 μL. Treatment groups were as follows: control (PBS, 0.5 mL weekly), ADI (5 IU, weekly), gemcitabine (125 mg/kg, two days per week) and combination of ADI and gemcitabine. Tumors were measured twice a week using calipers and volume was calculated by the following formula: *V* = (*L* × *W*)^2^*.* Animals were euthanized when tumors reached a diameter of 15 mm per institutional guidelines at which time tumors were removed and weighed. A representative tumor from each group was then fixed in 10% formalin and another tumor was frozen in liquid nitrogen. Statistical difference among tumor weights at sacrifice was determined by the Wilcoxon rank sum test with post-hoc Student’s t-test.

### Immunohistochemistry

Following formalin fixation, tumors were embedded in paraffin for subsequent immunohistochemistry. Slides were de-paraffinized and rehydrated through graded alcohols and rinsed with phosphate-buffered saline solution. Antigen retrieval was performed using the microwave technique in 10 mmol/L citrate buffer, pH 6. Primary antibodies included TUNEL SignalStain (K403-50, BioVision Inc., Mountain View, CA), and cleaved Caspase-3 (Asp175; Cell Signaling Technology, Inc., Denvers, MA). Visualization was performed using a biotinylated, streptavidin-HRP system (Vector Labs, Inc. Burlingame, CA) followed by diaminobenzidine. Sections were counterstained with Gill’s hematoxylin and fixed.

## Results

### PEG-ADI depletes intracellular arginine stores

**Figure 1:**
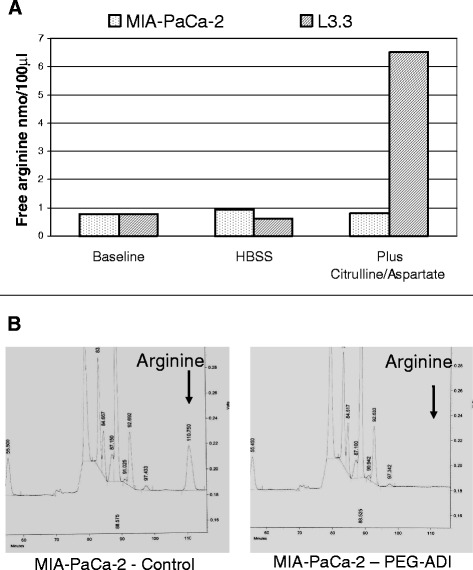
**PEG-ADI depletes extracellular arginine. A)** Levels of arginine (nmol/100 μl) in tissue culture media of MIA-PaCa-2 or L3.3 cells when cultured in complete media (baseline), following transition to HBSS, or HBSS supplemented with citrulline and aspartate; **B)** Levels of amino acids in tissue culture medium of exponentially growing MIA-PaCa-2 cells (left) and 24 hrs after treatment with PEG-ADI (1 μg/ml) (right) with arrow denoting HPLC peak of arginine.

We have previously shown that the L3.3 cell line expresses ASS and the MIA-PaCa-2 cell line is deficient in ASS [17]. To demonstrate that the MIA-PaCa-2 cell line has deficient arginine biosynthesis while this pathway is preserved in L3.3, cells were transitioned from complete growth medium to HBSS for 24 hours. Following this period of complete amino acid withdrawal, citrulline and aspartate (the substrates of arginine synthesis by ASS) were added for a period of four hours. MIA-PaCa-2 was unable to increase intracellular arginine levels, while L3.3 has a robust increase in intracellular arginine levels (Table 1, Figure 1A). We also wished to demonstrate that PEG-ADI depletes intracellular arginine in the ASS-deficient MIA-PaCa-2 cell line. Cells were cultured in the absence or presence of PEG-ADI (1.0 μg/ml) for 24 hours and intracellular amino acid content determined by HPLC. Intracellular arginine was undetectable following PEG-ADI treatment in these cells (Figure 1B).

**Table 1:** **Intracellular amino acid concentrations (nmol/100 μl) of L3.3 and MIA-PaCa-2 after a 24 hr incubation in HBSS, and 4 hours later in the absence or presence of exogenous citrulline and aspartate (1 mM)**

**L3.3**				
	**Baseline**	**T = 4 hrs (HBSS)**	**T = 4 hrs (+cit/asp)**	**Effect of cit/asp addition**
Citrulline	N.D.	N.D.	2.375	2.375
Arginine	0.778	0.624	6.511	5.887
Aspartate	7.565	6.310	5.141	−1.169
Ammonia	1.680	1.105	1.228	.123
Lysine	0.567	0.450	0.432	-.018
Phosphoserine	0.295	0.300	0.296	−0.004
AE-cys (int stand)	3.050	3.218	3.206	
**MIA-PaCa-2**				
	**Baseline**	**T = 4 hrs (HBSS)**	**T = 4 hrs (+cit/asp)**	**Change**
Citrulline	N.D.	N.D.	2.513	2.513
Arginine	0.768	0.949	0.802	−0.147
Aspartate	2.005	2.040	1.942	−0.098
Ammonia	4.316	4.162	4.338	.176
Lysine	0.537	0.618	0.572	-.046
Phosphoserine	0.261	0.219	0.189	−0.030
AE-cys (int stand)	3.188	3.311	3.206	

Lysine and phosphoserine are shown to demonstrate no other significant differences in amino acid levels, and AE-cys is an internal control spiked into every sample at 3 nmoles. N.D. = not detectable.

### In vitro interaction of PEG-ADI and gemcitabine

**Figure 2:**
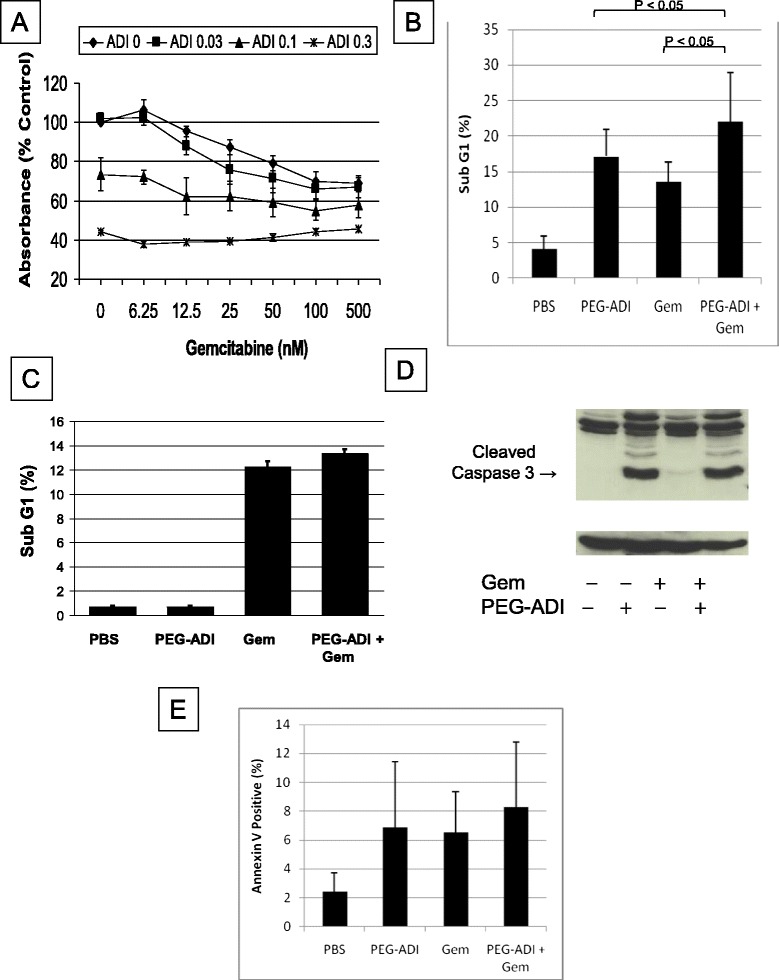
**Interaction of PEG-ADI and gemcitabine. A)** Dose-dependent effect of combining gemcitabine with PEG-ADI in MIA-PaCa-2 cells determined by MTT cytotoxicity assay, **B)** Effect of PEG-ADI, gemcitabine, or the combination on cell death measured by flow cytometry in MIA-PaCa-2 (mean of three separate experiments, +/- S.D), **C)** Effect of PEG-ADI, gemcitabine, or the combination on cell death measured by flow cytometry in L3.3 with no significant effect of PEG-ADI, either alone or in combination with gemcitabine, **D)** MIA-PaCa-2 cells treated with PEG-ADI, gemcitabine or the combination with immunoblotting for caspase 3 following gemcitabine, PEG-ADI or the combination with notation of cleaved caspase 3, **E)** Effect of PEG-ADI, gemcitabine or the combination on Annexin V staining of MIA-PaCa-2 cells.

We then sought to evaluate the interaction of PEG-ADI and gemcitabine on cell death in MIA-PaCa-2 cells, a human pancreatic cancer cell line relatively resistant to the cytotoxic effects of gemcitabine but sensitive to the effects of PEG-ADI [17,26]. A dose-dependent decrease in cell viability following gemcitabine treatment of MIA-PaCa-2 cells was observed, with an IC_50_ of approximately 500 nM, consistent with other reported studies (Figure 2A). PEG-ADI also demonstrated a dose-dependent decrease in cell viability, but when cells were treated with the combination, there was only a minor additive effect (Figure 2A). To confirm these findings, flow cytometry was performed to quantitate the apoptotic fraction of cells following these individual or combined treatments. The combination induced a slightly greater degree of apoptosis than either treatment alone (Figure 2B). In the L3.3 cell line, which is ASS-positive/PEG-ADI resistant, no cytotoxicity was observed following PEG-ADI treatment nor did it enhance the effect of gemcitabine (Figure 2C). To further examine the mode of cell death we analyzed the effect of combination therapy on markers of apoptosis. Gemcitabine does not induce strong caspase 3 cleavage when compared to PEG-ADI, and the combination of the two drugs does not increase caspase 3 cleavage beyond PEG-ADI treatment alone in MIA-PaCa-2 (Figure 2D). Because not all apoptosis is caspase dependent, we examined the effect of combination on an early marker of apoptosis, the loss of Annexin V positivity. Consistent with the caspase 3 cleavage findings, the combination therapy in MIA-PaCa-2 did not induce an increase in loss of Annexin V positivity (Figure 2E).

### E2F-1 represses RRM2 levels which is augmented by gemcitabine treatment

**Figure 3:**
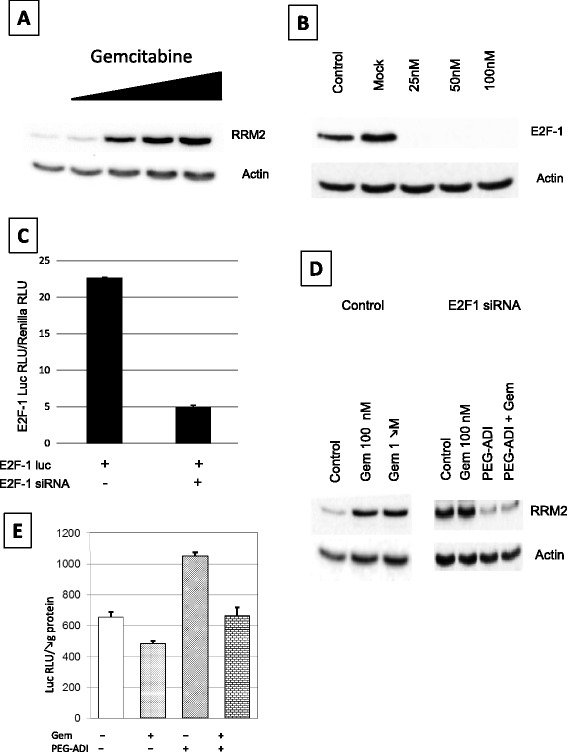
**Regulation of RRM2 by E2F-1. A)** Effect of increasing doses of gemcitabine on RRM2 levels in MIA-PaCa-2 cells, **B)** Effect of E2F-1 siRNA on E2F-1 protein levels in MIA-PaCa-2 cells, **C)** Effect of E2F-1 siRNA on E2F-1 transcription function in MIA-PaCa-2 cells determined by E2F-1-driven luciferase expression, **D)** Effect of E2F-1 siRNA on RRM2 levels following treatment with gemcitabine, PEG-ADI or the combination compared to the gemcitabine-mediated upregulation of RRM2, **E)** Effect of gemcitabine, PEG-ADI or the combination on E2F-1 transcription function in MIA-PaCa-2 cells determined by E2F-1-driven luciferase expression.

Utilizing the MIA-PaCa-2 cell line, a dose-dependent increase in RRM2 was seen following gemcitabine exposure (Figure 3A). To evaluate the potential role of E2F-1 in the regulation of RRM2, siRNA was used to knockdown protein levels. A dose-finding study demonstrated that 25 nM of E2F-1 siRNA was sufficient to significantly reduce E2F-1 protein levels (Figure 3B). To demonstrate a functional consequence of E2F-1 knockdown, cells were transfected with a E2F-1 luciferase reporter construct in the absence or presence of E2F-1 siRNA, which demonstrated an approximate 80% reduction of E2F-1 transcriptional activity (Figure 3C). To evaluate the role of E2F-1 in RRM2 regulation in the setting of gemcitabine exposure, MIA-PaCa-2 cells were treated with gemcitabine in the absence or presence of E2F-1 siRNA. Knockdown of E2F-1 increased RRM2 expression (Figure 3D), consistent with the known function of E2F-1 as a repressive transcriptional regulator of RRM2 observed in other cell lines. However, in the setting of E2F-1 knockdown, gemcitabine treatment did not increase RRM2 levels. Furthermore, arginine depletion through PEG-ADI treatment both inhibited RRM2 levels as well as blocked gemcitabine-mediated upregulation (Figure 3D, right panel). These data suggest that gemcitabine induces RRM2 expression by decreasing E2F-1 function, which decreases the inhibitory transcriptional function of E2F-1 on RRM2.

### PEG-ADI abrogates gemcitabine-induced up-regulation of RRM2

**Figure 4:**
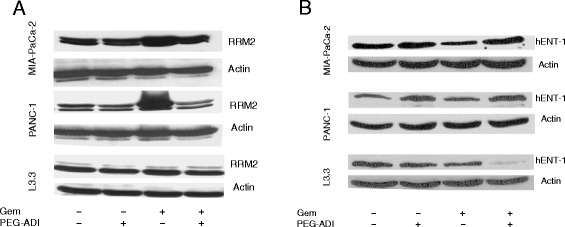
**PEG-ADI abrogates gemcitabine-mediated upregulation of RRM2 though E2F. A)** Effect of PEG-ADI, gemcitabine or the combination on RRM2 protein levels in MIA-PaCa-2, PANC-1 and L3.3 cells, **B)** Effect of PEG-ADI, gemcitabine or the combination on hENT-1 protein levels in MIA-PaCa-2, PANC-1 and L3.3 cells.

To demonstrate that the interaction of gemcitabine and PEG-ADI on RRM2, which we hypothesized is mediated by changes in activity of E2F-1, MIA-PaCa-2 cells were transfected with a E2F-luciferase reporter plasmid [25]. There was a decrease in E2F-1 activity following gemcitabine treatment; conversely, PEG-ADI increased E2F-1 activity. Most importantly, the repressive effect of gemcitabine on E2F-1 activity was blocked levels by simultaneous treatment with PEG-ADI (Figure 3E). These data suggest that PEG-ADI blocks gemcitabine-mediated upregulation of RRM2 through inhibition of the decrease in the repressive activity of E2F-1 activity following gemcitabine exposure. We then tested the effect of gemcitabine, PEG-ADI and the combination on four common mediators of gemcitabine resistance (RRM2, hENT-1, CDA and dCK) in three different pancreatic cancer cell lines with varying responsiveness to PEG-ADI. As noted previously, MIA-PaCa-2 is deficient in ASS expression, and undergoes arginine depletion following PEG-ADI treatment while L3.3 expresses ASS and maintains arginine levels following PEG-ADI exposure [17]. Gemcitabine induced significant upregulation of RRM2 in MIA-PaCa2 and PANC-1 cells (another ASS-deficient cell line), but concurrent treatment with PEG-ADI decreased RRM2 levels to baseline (Figure 4A). Interestingly, PEG-ADI had no discernible effect on RRM2 levels despite the prior observation that it could increase E2F-1 function, which we postulate is an inhibitor of RRM2 transcription. This effect was not seen in the ASS expressing cell line L3.3, as this cell line is resistant to any effect of PEG-ADI. When the effect of these treatments on hENT-1 levels was examined, only the combination of PEG-ADI and gemcitabine was noted to decrease hENT-1 levels in the ASS-expressing L3.3 cell line (Figure 4B). PEG-ADI did not affect the levels of dCK or CDA expression as a single agent or in combination with gemcitabine in MIA-PaCa-2, PANC-1 or L3.3 (data not shown).

### PEG-ADI synergistically enhances the tumor suppression effect of gemcitabine in vivo

**Figure 5:**
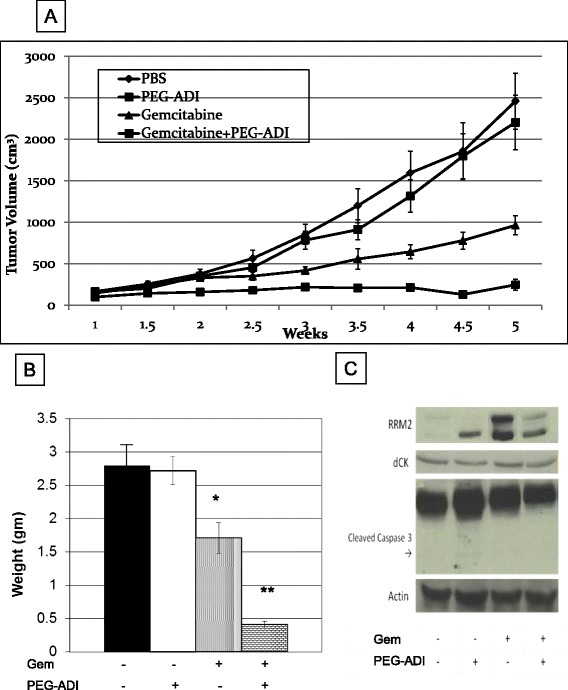
**PEG-ADI increases the efficacy of gemcitabine in vivo and is associated with abrogation of the gemcitabine-mediated induction of RRM2. A)** Mice with MIA-PaCa-2 xenografts were treated with PBS, PEG-ADI (5 IU, weekly), gemcitabine (125 mg/kg, twice weekly) or combination of PEG-ADI and gemcitabine. Tumor volumes were measured on indicated days and reported as mean ± SD, **B)** Weight of MIA-PaCa-2 xenografts (* = p < 0.05 vs. PEG-ADI; ** = p < 0.05 vs. gemcitabine), **C)** Lysates of the MIA-PaCa-2 xenografts with immunoblotting for RRM2, dCK or cleaved caspase 3 following the treatments noted.

To test the effect of combination treatment in vivo, mice bearing subcutaneous xenografts of MIA-PaCa2 were treated with PBS, PEG-ADI, gemcitabine or the combination of PEG-ADI and gemcitabine. PEG-ADI demonstrated little effect on tumor growth, while gemcitabine showed a modest tumor suppression activity (Figure 5A). The combination therapy, however, showed remarkable efficacy without any toxicity to the mice over a period of 7 weeks. Overall growth in the combination group was approximately 1/3 of that seen in the gemcitabine group and 1/6 of the PEG-ADI group. At time of animal sacrifice the average tumor weight in the combination group was 0.4 gm versus 1.7 gm in gemcitabine group and 2.7 gm in the PEG-ADI group (Figure 5B).

**Figure 6:**
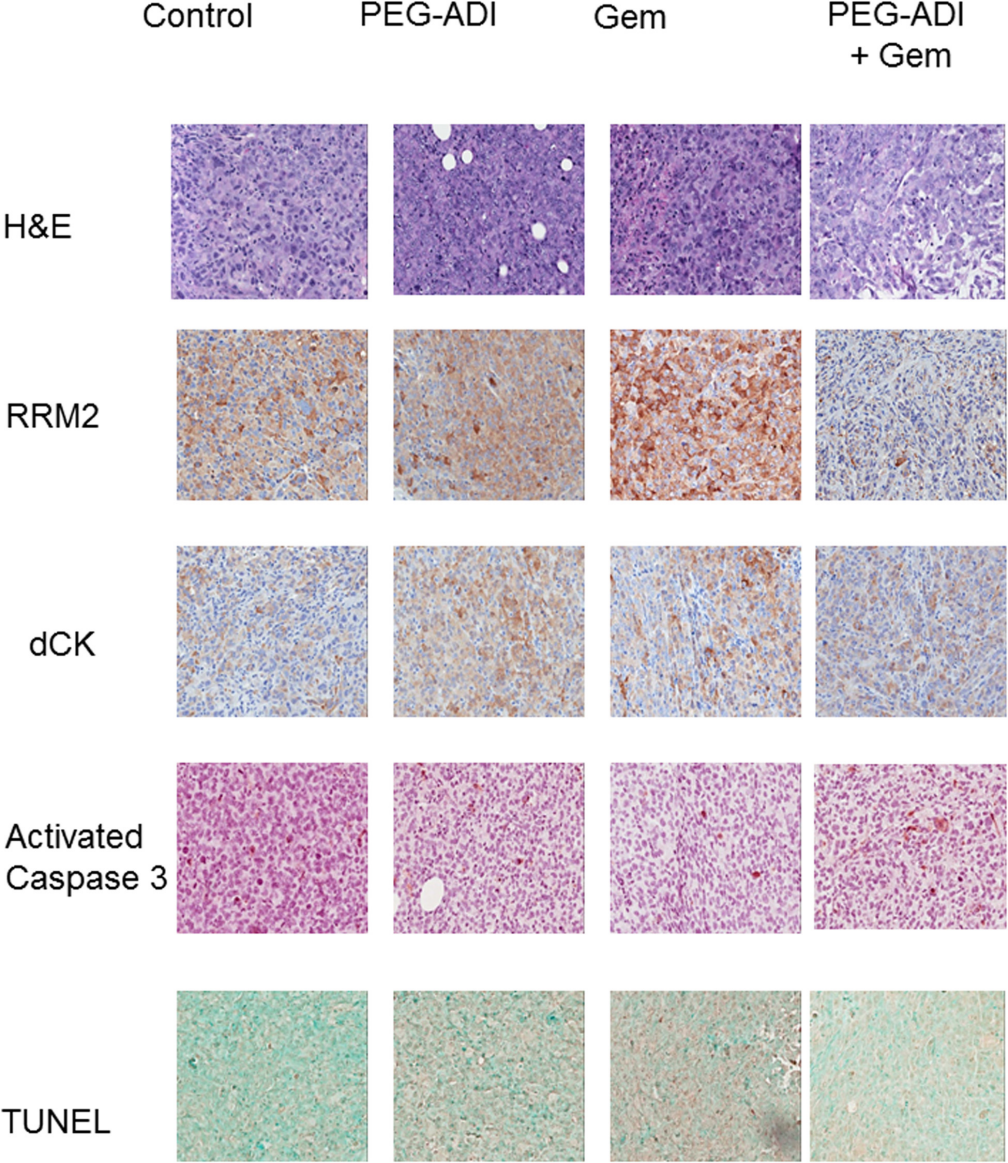
**Immunohistochemical staining of MIA-PaCa-2 xenografts.** MIA-PaCa-2 tumors treated with PBS (Control), ADI, gemcitabine or the combination (ADI + Gem) with immunohistochemical staining for RRM2, dCK, activated caspase 3 and TUNEL staining demonstrating the abrogation of gemcitabine-induced RRM2 with simultaneous ADI treatment. No treatment group had any significant effect on routine histology (H&E), dCK or cleaved caspase 3 levels or TUNEL staining.

At time of animal sacrifice tumors were preserved either by flash freezing in liquid nitrogen or fixed in 10% formalin. The mode of cell death was examined by immunblotting tumor lysates for activated caspase 3. No significant activation of the caspase cascade was observed in any of the treatments groups, in contrast to the in vitro data (Figure 5C). The effect of PEG-ADI on gemcitabine resistance markers dCK and RRM2 was also analyzed by immunoblotting. As observed in the in vitro studies, PEG-ADI did not affect dCK expression, but reduced the induction of RRM2 following gemcitabine treatment (Figure 5C). These finding were confirmed by immunohistochemical analysis of the formalin-fixed, paraffin-embedded tumors. Gemcitabine treatment significantly induced RRM2, and this induction was abrogated by simultaneous PEG-ADI treatment (Figure 6). Furthermore, no significant cytoplasmic cleaved caspase-3 was observed in the tumors treated with either PEG-ADI, gemcitabine or the combination. TUNEL staining also failed to demonstrate significant differences in apoptosis among any of the treatment groups. Significant necrosis was also absent as demonstrated by routine histologic staining.

## Discussion

**Figure 7:**
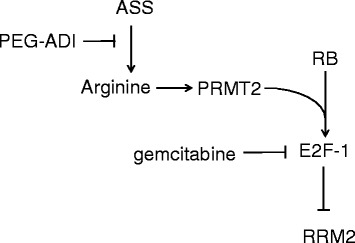
**Graphic representation of regulation of RRM2 by E2F-1.** Proposed signaling pathway of regulation of RRM2 by E2F-1 with subsequent upstream inhibition or stimulation by gemcitabine or arginine.

Despite the clearly cytotoxic effect of gemcitabine in vitro, clinical experience with its use in the treatment of PDAC shows only a modest cytostatic effect. In fact, gemcitabine is approved for palliation of PDAC based on clinical trials showing superior control of symptoms and only a modest increase in disease time-to-progression when compared to 5-fluorouracil. One explanation for the failure of gemcitabine to show objective tumor response is the high rates of inducible resistance in PDAC. Multiple pathways of gemcitabine resistance have been identified in PDAC. The two best characterized mechanisms of resistance in PDAC are the downregulation of deoxcytidine kinase (dCK) and induction of RRM2 [5–9,26,27]. Though PEG-ADI does not show an effect on expression of dCK, it shows a significant ability to abrogate the gemcitabine-mediated induction of RRM2. This effect appears to be mediated by regulation of E2F-1; gemcitabine suppresses E2F-1 activity leading to increased RRM2 whereas PEG-ADI increases E2F-1 function to prevent this potential mechanism of gemcitabine resistance (Figure 7). This mechanism is significant because reduction of RRM2 is not simply a marker of increased chemosensitivity, but a clinically relevant mechanism to overcome gemcitabine resistance. Duxbury and colleagues showed that knockdown of RRM2 by RNA interference significantly enhanced the cytotoxic effect of gemcitabine [10]. In vivo subcutaneous xenografts of MIA-PaCa2 showed significant tumor suppression when RRM2 siRNA was combined with gemcitabine treatment. Therefore, part of the ability of PEG-ADI to increase the response of human pancreatic cancer cells to gemcitabine may be through abrogation of the compensatory cellular response of RRM2 induction.

We noted significant conflicts in the in vitro data compared to the in vivo data. In both models, the combination of gemcitabine and PEG-ADI led to greater anti-tumor effect though the observed mechanism may be different. In cell culture experiments, short-term PEG-ADI induced a caspase-dependent apoptotic cell death. Although the combination of PEG-ADI with gemcitabine in vivo was associated with a significant anti-tumor response, very low levels of caspase cleavage or apoptosis were noted in all treatment groups. These data would suggest that the primary mechanism by which PEG-ADI increases the effect of gemcitabine in vivo is not related to the caspase-dependent apoptosis that can be observed with higher doses of PEG-ADI, which we have previously shown can inhibit pancreatic tumor growth as a monotherapy [17]. Furthermore, PEG-ADI did not have any significant effect on RRM2 levels in vitro (Figure 4A), though increased expression of one subunit in vivo (Figure 5C); this mechanism may involve regulation of other transcriptional mediators of RRM2 that are triggered by the local tumor environment that is not recapitulated in vitro. These differences between in vitro and in vivo are an important aspect of selection of therapies for combination based on potential synergy of mechanism for which we propose a lower dose of PEG-ADI for subsequent human clinical trials than would have been predicted on in vitro data alone.

Although PEG-ADI has been developed as a single agent therapy in hepatocellular carcinoma and melanoma [21,28,29], this approach may not be optimal for other solid organ malignancy. Although arginine deprivation has been shown in various models to induce both apoptotic and autophagic cell death associated with the auxotrophic effect of arginine deprivation [30,31], we propose that targeted arginine deprivation induces cellular changes that re-program cells allowing sensitization to traditional chemotherapy [32]. Arginine has diverse intracellular roles including nitric oxide (NO) formation, protein biosynthesis, and polyamine formation through ornithine generation. An example of the potentiating effect of arginine depletion was observed by Tsai et al who demonstrated that arginine depletion of melanoma cells down-regulated HIF-1α [33]. This effect was confirmed by Yoon et al in renal cell carcinoma in which PEG-ADI significantly reduced tumor angiogenesis and VEGF expression [34]. In these examples, the effect of arginine deprivation is not directly cancer cell toxicity, but a secondary effect altering response to subsequent therapy, such as radiation therapy [35]. Thus, similar to the experience of angiogenesis inhibitors, there have rarely been found to be effects as single agent therapy though efficacy is observed when combined with traditional cytotoxic therapies such as radiation or chemotherapy.

Arginine deprivation in pancreatic cancer cells auxotrophic for arginine appears to have multiple effects though we propose the primary mechanism in the chemosensitizing effect in combination with gemcitabine is through E2F-1-mediated regulation of RRM2 following gemcitabine exposure. The combination of PEG-ADI with gemcitabine in vivo yielded significant anti-tumor effects, with the benefit potentially involving the RRM2 induced gemcitabine resistance in PDAC. This implicates arginine deprivation as the likely mechanism by which PEG-ADI reduces RRM2 expression.

## Abbreviations

ASS: Argininosuccinate synthetase
hENT1: Human equilibrative nucleoside transporter-1
dCK: Deoxycytidine kinase
PDAC: Pancreatic ductal adenocarcinoma
PEG-ADI: Pegylated arginine deiminase
RB: Retinoblastoma
RRM2: Ribonucleotide reductase subunit M2
